# Enhancing search efficiency by means of a search filter for finding all studies on animal experimentation in PubMed

**DOI:** 10.1258/la.2010.009117

**Published:** 2010-07

**Authors:** Carlijn R Hooijmans, Alice Tillema, Marlies Leenaars, Merel Ritskes-Hoitinga

**Affiliations:** 1 Radboud University Nijmegen Medical Centre, Central Animal Laboratory and 3R Research Centre, Geert Grooteplein Noord 29, Route 231, 6525 EZ Nijmegen, The Netherlands; 2 Radboud University Nijmegen Medical Centre, Medical Library, Geert Grooteplein 15, Route 299, 6500 HB Nijmegen, The Netherlands

**Keywords:** Three Rs, ethics and welfare alternatives, ethics and welfare supplements to animal research, search filter, systematic review

## Abstract

Collecting and analysing all available literature before starting an animal experiment is important and it is indispensable when writing a systematic review (SR) of animal research. Writing such review prevents unnecessary duplication of animal studies and thus unnecessary animal use (Reduction). One of the factors currently impeding the production of ‘high-quality’ SRs in laboratory animal science is the fact that searching for *all* available literature concerning animal experimentation is rather difficult. In order to diminish these difficulties, we developed a search filter for PubMed to detect all publications concerning animal studies. This filter was compared with the method most frequently used, the PubMed *Limit: Animals*, and validated further by performing two PubMed topic searches. Our filter performs much better than the PubMed limit: it retrieves, on average, 7% more records. Other important advantages of our filter are that it also finds the most recent records and that it is easy to use. All in all, by using our search filter in PubMed, all available literature concerning animal studies on a specific topic can easily be found and assessed, which will help in increasing the scientific quality and thereby the ethical validity of animal experiments.

In a systematic review (SR), all available literature about a specific research question is identified, appraised, selected and ultimately extracted in order to generate new data. These new results provide scientists with a better understanding and an evidence-based summary of the present situation concerning the particular research question. SRs are regarded as the highest level of medical evidence by evidence-based medicine professionals. Although performing SRs is becoming standard practice in clinical studies, this is not yet the case for animal experiments.^1^

Executing an SR on literature on animal experiments will improve the interpretation of scientific results that have already been published. As a consequence, scientific quality will improve and patient safety will be optimized.^1^ In addition, using all the literature about a specific topic in an SR before starting a new experiment prevents unnecessary duplication of animal studies and thus unnecessary animal use (Reduction^2^). Moreover, a complete literature review will ensure that all relevant elements related to Reduction, Refinement and Replacement are taken into account and implemented. For these reasons, SRs ought to become standard practice when performing animal experiments.

It is currently rather difficult to perform ‘high-quality’ SRs in laboratory animal science. Not only because most papers do not report necessary details or are of poor scientific quality,^3–7^ but also because searching *all* available literature concerning animal experimentation is anything but simple. Although PubMed contains most of the papers concerning medicine-related animal experimentation, finding them all is a challenge. First of all, because most scientists do not know how to use PubMed efficiently. Our experience from tutorials on searching for information on animal experiments is that many researchers do not use ‘Medical Subject Headings’ (MeSH terms), even though they work with PubMed every day. MeSH is the National Library of Medicine's (NLM) controlled vocabulary thesaurus. It consists of sets of terms in a hierarchical structure, which permits searching at various levels of specificity. The MeSH database also provides entry terms to assist in finding the most appropriate MeSH heading; for example, ‘Vitamin C’ is an entry term to the MeSH term ‘Ascorbic Acid’.

Secondly, the NLM indexing process is sometimes unpredictable and may contain errors. For example, some studies get solely assigned the MeSH term *Mice*, instead of *Animals* and *Mice*. The consequence is that a search using the term *Animals* will not retrieve the studies solely assigned the MeSH term *Mice*.

Thirdly, the most recently submitted papers have not been indexed yet, and thus have no MeSH terms. As the currently available option for searching all studies on laboratory animals (the PubMed *Limit: Animals*) is based on indexing and is actually limiting a search to all records that have been assigned the MeSH term *Animals*, all recent studies will be missed. Moreover, studies that never received any MeSH terms will also not be found. To solve this problem, not only all relevant MeSH terms, but also all relevant search terms relating to the different animal species need to be included in the search strategy.

Fourthly, there is a large variation in terminology for all these different species. Authors use different spelling, use various synonyms for the same species or mention the species term only in the singular or the plural.

All of the above is illustrated in the next example about mice: ‘mice[tiab = title and/or abstract] OR mus[tiab] OR mouse[tiab] OR murine[tiab] OR wood mouse[tiab] etc.’.

Fifthly, when searching for animal studies, excluding human studies in a topic search is not advisable, because it is our experience that studies in which both humans and animals have been used will not be found.

In summary, there is a clear need for an effective search strategy in PubMed in order to find all studies concerning animal experimentation. The aim of our current study was to develop a complete search filter for PubMed in order to detect all publications on laboratory animals. In order to validate our search filter, we compared it with the most frequently used alternative for searching studies on animals in PubMed, the *Limit: Animals*.

## Methods

The search filter for finding studies on laboratory animals described in this paper was developed by scientists with extensive experience in laboratory animal science together with experts from the Medical Library of the Radboud University Nijmegen, The Netherlands.

### Search term selection and combination

**Figure 1:**
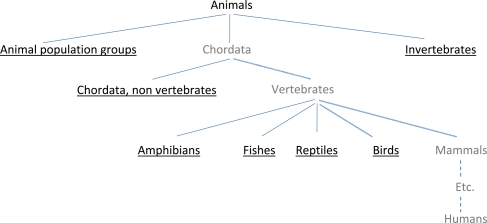
Medical Subject Headings (MeSH terms) are arranged hierarchically by subject category in the MeSH Tree, listing the more specific (narrower) terms beneath the more general (broader) terms. The black underlined terms are exploded in the search filter for laboratory animals, the grey terms are not exploded

Search terms were identified by using the annual report of the Food and Consumer Product Safety Authority in The Netherlands (VWA – Dutch Inspectorate for animal experimentation)^8^ and the fifth report from the Commission to the Council and the European Parliament on the statistics on the number of animals used for experimental and other scientific purposes in the member states of the European Union (COM/2007/675 final^9^). The annual report of the VWA contains information about all the animal experiments performed at licensed institutes in The Netherlands and provided an overview of the animal species used in animal experimentation in The Netherlands. The fifth report from the Commission to the Council and the European Parliament was used in order to get an overview of the laboratory animal species used in other European countries. Subsequently, we identified relevant MeSH terms in the MeSH database and included all relevant terms in our filter both as a MeSH term and as a single word or phrase in the title and/or abstract [tiab]. The MeSH terms are arranged hierarchically by subject category in the MeSH Tree, listing the more specific (narrower) terms below the more general (broader) terms (see Figure 1). ‘Exploding a MeSH term’ means that its narrower terms are included in the search strategy as well. In our current search filter, almost all MeSH terms were exploded, except for the subject categories/branches that contain the MeSH term ‘humans’ as a specific narrower term somewhere in the MeSH hierarchy. The latter MeSH terms were added with the option ‘Do Not Explode this term’ (‘no exp’; the grey terms in Figure 1). All the subcategories belonging to these terms but *not* including ‘humans’ were added to the filter *with* ‘explosion’ (the black underlined terms in Figure 1).

The first part of the search filter consists of all MeSH terms in the subject category *Animals* combined with OR, either with or without explosion of the term as explained above.

For the second part of the filter, again the operator OR was used to combine all entry terms mentioned in the MeSH database in the title and/or abstract [tiab]. Next, terms based on our own expertise were added in the title and/or abstract. Last but not least, all relevant search terms were listed in all their specific forms (i.e. singular, plural, Latin, American English and British English).

The second part contains all relevant terms in [tiab] combined with the command *NOT medline*[*sb = subset*] in order to make sure that this part of the search only retrieves terms in the title and/or abstract of records that are not indexed for Medline and thus do not have any MeSH terms. This approach excludes all records indexed with MeSH terms that are different from the ones mentioned in the first part of the filter, especially the MeSH term *Humans*. The second part of the filter is built in order to find all the recent papers not yet indexed for Medline.

### Application of the search filter

The search filter is an elaborate enumeration of relevant search terms combined with Boolean operators. The search strategy will be electronically available as Supplementary data, in order to facilitate easy use by anyone who is trying to identify all available studies on laboratory animals in PubMed in their own field. A possible approach would be to paste an electronic copy of the search filter into the search box of PubMed (www.ncbi.nlm.nih.gov/pubmed/) and press *Search*. The search will then be available as a separate search number from the Search History on the Advanced Search page. After that any topic search may be combined with the search filter using AND so as to determine the number of animal studies on that particular topic. An account with My NCBI offers the option of creating and saving custom search filters to simplify the narrowing down of search results for every single search without having to execute a combination of searches first. In the right column on the results page of PubMed the number of records per filter will be visible under ‘Filter your results’.

### Evaluation and validation of the search filter

Our newly developed search filter for detecting all studies in PubMed in which laboratory animals are used or described was compared with the easily available and most obvious method (‘regular method’), the PubMed *Limit: Animals*. The number of records obtained with the two different methods was compared. In addition, both the search filter and the limit option were validated by actually performing two PubMed topic searches. The first topic search aimed at finding all available literature in PubMed about probiotic use in experimental pancreatitis (Supplement 1a, available online at http:la.rsmjournals.com/cgi/content/full/la.2010.009117/DC1 ).

The second topic search tried to identify all studies about food restriction in laboratory animals (Supplement 1b, available online at http:la.rsmjournals.com/cgi/content/full/la.2010.009117/DC1 ).

The number of records found with our search filter as a proportion of the number of records retrieved by the *Limit: Animals* was calculated. We will refer to this proportion as the sensitivity* of the search filter.

## Results

The search filter for finding all PubMed studies on laboratory animals is presented in Table 1, which can also be found in the online supplementary material at http:la.rsmjournals.com/cgi/content/full/la.2010.009117/DC1

**Table 1:** PubMed search filter for laboratory animals

First part	("animal experimentation"[MeSH Terms] OR "models, animal"[MeSH Terms] OR "invertebrates"[MeSH Terms] OR "Animals"[Mesh:noexp] OR "animal population groups"[MeSH Terms] OR "chordata"[MeSH Terms:noexp] OR "chordata, nonvertebrate"[MeSH Terms] OR "vertebrates"[MeSH Terms:noexp] OR "amphibians"[MeSH Terms] OR "birds"[MeSH Terms] OR "fishes"[MeSH Terms] OR "reptiles"[MeSH Terms] OR "mammals"[MeSH Terms:noexp] OR "primates"[MeSH Terms:noexp] OR "artiodactyla"[MeSH Terms] OR "carnivora"[MeSH Terms] OR "cetacea"[MeSH Terms] OR "chiroptera"[MeSH Terms] OR "elephants"[MeSH Terms] OR "hyraxes"[MeSH Terms] OR "insectivora"[MeSH Terms] OR "lagomorpha"[MeSH Terms] OR "marsupialia"[MeSH Terms] OR "monotremata"[MeSH Terms] OR "perissodactyla"[MeSH Terms] OR "rodentia"[MeSH Terms] OR "scandentia"[MeSH Terms] OR "sirenia"[MeSH Terms] OR "xenarthra"[MeSH Terms] OR "haplorhini"[MeSH Terms:noexp] OR "strepsirhini"[MeSH Terms] OR "platyrrhini"[MeSH Terms] OR "tarsii"[MeSH Terms] OR "catarrhini"[MeSH Terms:noexp] OR "cercopithecidae"[MeSH Terms] OR "hylobatidae"[MeSH Terms] OR "hominidae"[MeSH Terms:noexp] OR "gorilla gorilla"[MeSH Terms] OR "pan paniscus"[MeSH Terms] OR "pan troglodytes"[MeSH Terms] OR "pongo pygmaeus"[MeSH Terms])
Second part	OR ((animals[tiab] OR animal[tiab] OR mice[Tiab] OR mus[Tiab] OR mouse[Tiab] OR murine[Tiab] OR woodmouse[tiab] OR rats[Tiab] OR rat[Tiab] OR murinae[Tiab] OR muridae[Tiab] OR cottonrat[tiab] OR cottonrats[tiab] OR hamster[tiab] OR hamsters[tiab] OR cricetinae[tiab] OR rodentia[Tiab] OR rodent[Tiab] OR rodents[Tiab] OR pigs[Tiab] OR pig[Tiab] OR swine[tiab] OR swines[tiab] OR piglets[tiab] OR piglet[tiab] OR boar[tiab] OR boars[tiab] OR "sus scrofa"[tiab] OR ferrets[tiab] OR ferret[tiab] OR polecat[tiab] OR polecats[tiab] OR "mustela putorius"[tiab] OR "guinea pigs"[Tiab] OR "guinea pig"[Tiab] OR cavia[Tiab] OR callithrix[Tiab] OR marmoset[Tiab] OR marmosets[Tiab] OR cebuella[Tiab] OR hapale[Tiab] OR octodon[Tiab] OR chinchilla[Tiab] OR chinchillas[Tiab] OR gerbillinae[Tiab] OR gerbil[Tiab] OR gerbils[Tiab] OR jird[Tiab] OR jirds[Tiab] OR merione[Tiab] OR meriones[Tiab] OR rabbits[Tiab] OR rabbit[Tiab] OR hares[Tiab] OR hare[Tiab] OR diptera[Tiab] OR flies[Tiab] OR fly[Tiab] OR dipteral[Tiab] OR drosphila[Tiab] OR drosophilidae[Tiab] OR cats[Tiab] OR cat[Tiab] OR carus[Tiab] OR felis[Tiab] OR nematoda[Tiab] OR nematode[Tiab] OR nematoda[Tiab] OR nematode[Tiab] OR nematodes[Tiab] OR sipunculida[Tiab] OR dogs[Tiab] OR dog[Tiab] OR canine[Tiab] OR canines[Tiab] OR canis[Tiab] OR sheep[Tiab] OR sheeps[Tiab] OR mouflon[Tiab] OR mouflons[Tiab] OR ovis[Tiab] OR goats[Tiab] OR goat[Tiab] OR capra[Tiab] OR capras[Tiab] OR rupicapra[Tiab] OR chamois[Tiab] OR haplorhini[Tiab] OR monkey[Tiab] OR monkeys[Tiab] OR anthropoidea[Tiab] OR anthropoids[Tiab] OR saguinus[Tiab] OR tamarin[Tiab] OR tamarins[Tiab] OR leontopithecus[Tiab] OR hominidae[Tiab] OR ape[Tiab] OR apes[Tiab] OR pan[Tiab] OR paniscus[Tiab] OR "pan paniscus"[Tiab] OR bonobo[Tiab] OR bonobos[Tiab] OR troglodytes[Tiab] OR "pan troglodytes"[Tiab] OR gibbon[Tiab] OR gibbons[Tiab] OR siamang[Tiab] OR siamangs[Tiab] OR nomascus[Tiab] OR symphalangus[Tiab] OR chimpanzee[Tiab] OR chimpanzees[Tiab] OR prosimians[Tiab] OR "bush baby"[Tiab] OR prosimian[Tiab] OR bush babies[Tiab] OR galagos[Tiab] OR galago[Tiab] OR pongidae[Tiab] OR gorilla[Tiab] OR gorillas[Tiab] OR pongo[Tiab] OR pygmaeus[Tiab] OR "pongo pygmaeus"[Tiab] OR orangutans[Tiab] OR pygmaeus[Tiab] OR lemur[Tiab] OR lemurs[Tiab] OR lemuridae[Tiab] OR horse[Tiab] OR horses[Tiab] OR pongo[Tiab] OR equus[Tiab] OR cow[Tiab] OR calf[Tiab] OR bull[Tiab] OR chicken[Tiab] OR chickens[Tiab] OR gallus[Tiab] OR quail[Tiab] OR bird[Tiab] OR birds[Tiab] OR quails[Tiab] OR poultry[Tiab] OR poultries[Tiab] OR fowl[Tiab] OR fowls[Tiab] OR reptile[Tiab] OR reptilia[Tiab] OR reptiles[Tiab] OR snakes[Tiab] OR snake[Tiab] OR lizard[Tiab] OR lizards[Tiab] OR alligator[Tiab] OR alligators[Tiab] OR crocodile[Tiab] OR crocodiles[Tiab] OR turtle[Tiab] OR turtles[Tiab] OR amphibian[Tiab] OR amphibians[Tiab] OR amphibia[Tiab] OR frog[Tiab] OR frogs[Tiab] OR bombina[Tiab] OR salientia[Tiab] OR toad[Tiab] OR toads[Tiab] OR "epidalea calamita"[Tiab] OR salamander[Tiab] OR salamanders[Tiab] OR eel[Tiab] OR eels[Tiab] OR fish[Tiab] OR fishes[Tiab] OR pisces[Tiab] OR catfish[Tiab] OR catfishes[Tiab] OR siluriformes[Tiab] OR arius[Tiab] OR heteropneustes[Tiab] OR sheatfish[Tiab] OR perch[Tiab] OR perches[Tiab] OR percidae[Tiab] OR perca[Tiab] OR trout[Tiab] OR trouts[Tiab] OR char[Tiab] OR chars[Tiab] OR salvelinus[Tiab] OR "fathead minnow"[Tiab] OR minnow[Tiab] OR cyprinidae[Tiab] OR carps[Tiab] OR carp[Tiab] OR zebrafish[Tiab] OR zebrafishes[Tiab] OR goldfish[Tiab] OR goldfishes[Tiab] OR guppy[Tiab] OR guppies[Tiab] OR chub[Tiab] OR chubs[Tiab] OR tinca[Tiab] OR barbels[Tiab] OR barbus[Tiab] OR pimephales[Tiab] OR promelas[Tiab] OR "poecilia reticulata"[Tiab] OR mullet[Tiab] OR mullets[Tiab] OR seahorse[Tiab] OR seahorses[Tiab] OR mugil curema[Tiab] OR atlantic cod[Tiab] OR shark[Tiab] OR sharks[Tiab] OR catshark[Tiab] OR anguilla[Tiab] OR salmonid[Tiab] OR salmonids[Tiab] OR whitefish[Tiab] OR whitefishes[Tiab] OR salmon[Tiab] OR salmons[Tiab] OR sole[Tiab] OR solea[Tiab] OR "sea lamprey"[Tiab] OR lamprey[Tiab] OR lampreys[Tiab] OR pumpkinseed[Tiab] OR sunfish[Tiab] OR sunfishes[Tiab] OR tilapia[Tiab] OR tilapias[Tiab] OR turbot[Tiab] OR turbots[Tiab] OR flatfish[Tiab] OR flatfishes[Tiab] OR sciuridae[Tiab] OR squirrel[Tiab] OR squirrels[Tiab] OR chipmunk[Tiab] OR chipmunks[Tiab] OR suslik[Tiab] OR susliks[Tiab] OR vole[Tiab] OR voles[Tiab] OR lemming[Tiab] OR lemmings[Tiab] OR muskrat[Tiab] OR muskrats[Tiab] OR lemmus[Tiab] OR otter[Tiab] OR otters[Tiab] OR marten[Tiab] OR martens[Tiab] OR martes[Tiab] OR weasel[Tiab] OR badger[Tiab] OR badgers[Tiab] OR ermine[Tiab] OR mink[Tiab] OR minks[Tiab] OR sable[Tiab] OR sables[Tiab] OR gulo[Tiab] OR gulos[Tiab] OR wolverine[Tiab] OR wolverines[Tiab] OR minks[Tiab] OR mustela[Tiab] OR llama[Tiab] OR llamas[Tiab] OR alpaca[Tiab] OR alpacas[Tiab] OR camelid[Tiab] OR camelids[Tiab] OR guanaco[Tiab] OR guanacos[Tiab] OR chiroptera[Tiab] OR chiropteras[Tiab] OR bat[Tiab] OR bats[Tiab] OR fox[Tiab] OR foxes[Tiab] OR iguana[Tiab] OR iguanas[Tiab] OR xenopus laevis[Tiab] OR parakeet[Tiab] OR parakeets[Tiab] OR parrot[Tiab] OR parrots[Tiab] OR donkey[Tiab] OR donkeys[Tiab] OR mule[Tiab] OR mules[Tiab] OR zebra[Tiab] OR zebras[Tiab] OR shrew[Tiab] OR shrews[Tiab] OR bison[Tiab] OR bisons[Tiab] OR buffalo[Tiab] OR buffaloes[Tiab] OR deer[Tiab] OR deers[Tiab] OR bear[Tiab] OR bears[Tiab] OR panda[Tiab] OR pandas[Tiab] OR "wild hog"[Tiab] OR "wild boar"[Tiab] OR fitchew[Tiab] OR fitch[Tiab] OR beaver[Tiab] OR beavers[Tiab] OR jerboa[Tiab] OR jerboas[Tiab] OR capybara[Tiab] OR capybaras[Tiab]) NOT medline[subset])

The performance of the search filter was analysed against the most regular method, the PubMed *Limit: Animals*. Table 2 shows that with the PubMed limit 4,411,585 records were found, whereas our search filter retrieved 4,689,950 records. All the records found with the PubMed limit were also included in the search result of our filter (Table 2; #3). The search filter (part 1 plus part 2) found 278,365 records more than the PubMed limit. The sensitivity* of the filter here is 4,689,950/4,411,585 × 100% = 106.3%.

**Table 2:** Results of literature searches in PubMed using the ‘regular method’ (PubMed *Limit*: *Animals*) and our search filter (performed on 29 September 2009)

Search	Query	Results
#1	PubMed *Limit: Animals*	4,411,585
#2	Our search filter	4,689,950
#3	#1 AND #2	4,411,585
#4	#2 NOT #1	278,365
#5	MeSH part of our search filter	4,556,355
#6	#5 NOT #1	144,770
#7	#2 NOT #5	133,595
#8	(probiotics AND pancreatitis) AND #1	33
#9	(probiotics AND pancreatitis) AND #2	37
#10	#9 NOT #8	4
#11	Food restriction AND #1	9280
#12	Food restriction AND #2	9650
#13	#12 NOT #11	370

AND: overlay, records present in both searches; NOT: records present in one search but not in the other

Since the PubMed *Limit: Animals* searches solely for the MeSH term *Animals* without explosion (‘Animals’[MeSH Terms:noexp] = #1), we compared this PubMed limit with the first part of the newly developed search filter, which solely contains MeSH terms (#5). The MeSH part of the search filter yielded 144,770 records more than the PubMed *Limit: Animals*. Of the total number of extra records found with the complete search filter (part 1 plus part 2; #4), 52% were records with MeSH terms (#6), whereas 48% turned out to be records without any MeSH terms (#7).

In order to highlight the role of the search filter in developing SRs in laboratory animal science, the search filter and the ‘regular method’ were also validated by performing two PubMed topic searches (Table 2). The first topic search on the use of probiotics in experimental pancreatitis retrieved 33 items with the *Limit: Animals*, whereas this strategy combined with the new search filter retrieved 37 items. The sensitivity* in this search strategy was therefore 112.1%.

The second topic search aimed at the identification of all studies dealing with food restriction in laboratory animals and retrieved 9280 records with the *Limit: Animals*, whereas this strategy combined with our complete filter retrieved 9650 items. The sensitivity* in this search strategy was therefore 104%.

All in all, it can be concluded that our search filter retrieves 7% more records as compared with the regular method (*Limit: Animals*) in PubMed, since a mean sensitivity* of 107% was found.

## Discussion

Scientists need to be able to find all literature about laboratory animals when preparing or executing animal experiments and when writing an SR about their topic. In this paper, we have presented a search filter for finding all records on laboratory animal studies in PubMed. This search filter has been compared with the regular search method in PubMed, namely using the *Limit: Animals*.

Up to now, it has proven to be difficult to find all the different records about laboratory animal use in the PubMed database. This is not only because the NLM indexing process is sometimes unpredictable and most scientists do not know how to use PubMed effectively, but also because the most recently submitted papers have not been indexed yet and consequently can only be found by using search terms in the title and/or abstract [tiab]. Moreover, there are many different animal species used in laboratory animal science and there is a large variation in terminology for all these different species. To diminish these problems significantly, we have developed a search filter for finding all records on laboratory animals in PubMed. We used the annual report of the Food and Consumer Product Safety Authority in The Netherlands (VWA)^8^ and the fifth report from the Commission to the Council and the European Parliament on the statistics on the number of animals used for experimental and other scientific purposes in the member states of the European Union (COM/2007/675 final) to determine the different animal species employed in animal experimentation in Europe. Although we assume that largely the same species are used in the laboratories in the rest of the world, species used only in particular non-European countries might be missing from our filter. Scientists who want to use this filter are invited to adapt it to their own specific needs.

Despite the possible omissions just mentioned, our filter performs much better than the current alternative in PubMed, the *Limit: Animals*: our filter retrieves 7% more records concerning animals. There are two main explanations for this difference. First, unlike the PubMed *Limit: Animals*, we have exploded all the relevant animal-related MeSH terms from the MeSH hierarchical tree. By using the *Limit: Animals*, solely the records to which the MeSH term *Animals* has been assigned are found, whereas our search filter also retrieves all records to which more specific MeSH terms like *Mice* have been assigned but not the MeSH term *Animals*.

Secondly, the papers published most recently have not yet been indexed by Medline and therefore are not found by using the PubMed *Limit: Animals*. The same applies to papers that have never been indexed by Medline. The filter presented in this paper solves this problem by including all relevant search terms for different animal species in [tiab] in the search strategy.

So, a major advantage of this search filter is that it also finds the most recent records. Other important advantages are that the search filter searches for all synonyms of all different laboratory animal species at once and that it is easy to use (copy and paste the search strategy into the search box of PubMed and run it). To our knowledge, there is currently no other search strategy available with the same advantages. Some researchers make use of the search string ‘NOT ((humans) NOT (humans AND animals))’ in order to exclude studies purely about humans and to include studies about animals AND humans (for full phrase: Supplement 2, available online at http:la.rsmjournals.com/cgi/content/full/la.2010.009117/DC1 ). However, this search string excludes all records in which a specific animal species is mentioned in the title or abstract, but not the search term animal. In addition, compared with our search filter, this search string is less specific.

Our search filter also has some limitations. First of all, this complete search filter for laboratory animals is only suitable for use in PubMed. A search filter for other databases, like Embase and ISI Web of Science, is needed as well, particularly when writing an SR, since the guidelines require an extensive search in at least two different databases. The second part of the search filter, containing all the search terms in [tiab] (without the NOT medline[sb] phrase), can be used in Embase and ISI Web of Science as well, but the first part of the filter containing all the thesaurus terms is specific to PubMed.

Secondly, the filter does not make a distinction between records in which animals are mentioned, but that are not really about laboratory animals or animal experiments, and records in which animals are the major subject.

Generally, the performance of search filters is evaluated by calculating sensitivity (number of relevant records retrieved by the search filter as a proportion of the total number of relevant records) and specificity (number of irrelevant records NOT retrieved by the search filter as a proportion of the total number of irrelevant records).^10^ In the case of our filter, however, it would be very difficult to determine the total number of relevant and irrelevant records. All records in (large subsets of) PubMed would have to be collected and their relevance would have to be determined ‘by hand’. Moreover, the relevance of a record is partly dependent on the specific research question (yielded studies about bird flu in humans might be irrelevant when investigating bird flu in birds, but might be relevant when investigating the effects of bird flu in different species including humans). In light of these difficulties and given the aim of our filter, we decided to validate the filter by comparing it with the only alternative currently available, the PubMed *Limit: Animals*, and calculate sensitivity relative to this alternative (what we called sensitivity*). Although we do not, for the reasons just mentioned, make quantitative statements about the sensitivity and specificity of our filter, we will give some qualitative comments.

Our PubMed search filter identifies all records in which laboratory animals are mentioned or have been used directly or indirectly, and consequently a very broad variety of papers will be retrieved. For example, reviews, comments, original papers about animal experimentation and studies with tissues originating from animals will all be found. The sensitivity of the filter is therefore likely to be high and may even be 100%. The specificity of the filter, on the other hand, will probably be far lower since irrelevant studies will, as a result of the design of the filter, certainly also be found. For example, studies concerning swine flu that have nothing to do with pigs or studies mentioning the term ‘animal model’ somewhere in the abstract in order to conclude that there is no animal model available in their research field.

In order to make sure that our filter does not retrieve an excessive number of irrelevant records (in other words, to increase specificity), we have added the phrase ‘NOT medline[sb]’ to the [tiab] part of the search filter. This phrase ensures that PubMed is looking for terms only in the title and abstract of records in the database that are not indexed for Medline (and thus have no MeSH terms). The first part of the search filter (the MeSH terms) already retrieves all relevant papers indexed for Medline, whereas the second part of the filter only retrieves records with relevant terms in the title and/or abstract without the records that are indexed with irrelevant MeSH terms. For example, this approach excludes the ‘humans-only records’ with the MeSH term ‘humans’ and ‘bird flu’ in the title and/or abstract.

Because this search filter is very sensitive and thus retrieves numerous records, the filter may seem impractical. This would be the case if the filter were used on its own, but as a matter of fact a search strategy always consists of different search components (of which ‘laboratory animals’ is only one component) and the correct combination of these search components will decrease the number of records considerably. The examples in Table 2 show that a search with only the search filter retrieved 4.7 million records, whereas a complete and specific search strategy (consisting of three components: probiotics, pancreatitis and experimental animals) reduced the number of records to 37.

In summary, the PubMed search filter for laboratory animals presented in this paper was developed because there is a huge need for an effective search strategy in PubMed for finding all studies concerning the use of laboratory animals. It is very important to retrieve all relevant literature in order to prepare and execute an animal experiment in an optimal way and guarantee implementation of the three Rs.

SRs on animal experimentation are not yet standard practice; however, they should become the standard before starting a new project in which animal studies are going to be executed. An SR of animal studies is also needed before the start of clinical trials in order to guarantee patient safety. By using our effective search filter in PubMed, all available literature concerning a specific topic can be found and read, which will help in making better evidence-based decisions and result in optimal experimental conditions for both science and animal welfare.
